# Functional and Radiological Outcomes of Unstable Proximal Femur Fractures Fixed With Anatomical Proximal Locking Compression Plate

**DOI:** 10.7759/cureus.24903

**Published:** 2022-05-11

**Authors:** Vamsee Krishna, Aakaash Venkatesan, Ayush Kumar Singh

**Affiliations:** 1 Orthopedics, Sundaram Medical Foundation, Chennai, IND; 2 Orthopedics, Gaurav Orthopedic Hospital, Kolar, IND; 3 Trauma and Orthopedics, Worthing Hospital, Sussex, GBR; 4 Orthopedics, Cardiff and Vale University Health Board, Cardiff, GBR

**Keywords:** outcomes, harris hip score, unstable fractures, plating, peri-trochanteric

## Abstract

Introduction

Peritrochanteric fractures are the most frequent fractures of the proximal femur that accounts for nearly half of all proximal femur fractures. They are a major cause of disability in the elderly. The aim is to study the functional and radiological outcome of unstable proximal femur fractures fixed with proximal femur locking compression plate (PF-LCP) and its complications. Unstable proximal femur fracture patients operated with proximal femur locking compression plate were followed up functionally by Harris Hip Score and radiologically by neck-shaft angle measure.

Materials and methods

A retrospective analysis of 30 patients with unstable peritrochanteric fractures treated with PF-LCP in the first-level trauma center was conducted between 2015 and 2019. Stable peritrochanteric, pediatric and open fractures, and polytrauma were excluded. As a mid-term follow-up, functional and radiological outcomes were assessed at six weeks, three months, six months, and 12 months. Data was analyzed using a chi-square test, and results were compared with available western literature.

Results

Thirty patients with unstable peritrochanteric fractures operated between 2015 and 2019, complying with our inclusion criteria, were analyzed. All patients were operated by the same surgeon and were available for a mid-term follow-up (12 months). Mean radiological union time was 12.5+/-2 weeks, with 24 patients achieving union between 10-15 weeks, three patients had union little more than 15 weeks. Two patients had non-union and required re-surgery. Functional results were assessed in the 30 patients available for follow-up using Harris Hip Score. Excellent results were seen in 17, good in seven, fair in three, and poor in three patients.

Conclusions

The choice of implant used to manage unstable peritrochanteric fractures has always been a debatable subject in our orthopedic fraternity. In our study, we used the anatomic, fixed-angle plates* *in peritrochanteric fractures and obtained significant functional and radiological outcomes over a midterm follow-up. We recommend PF-LCP as a good, stable alternative in the treatment of peritrochanteric femoral fractures. We consider that fracture pattern and extent in the proximal femur have a definite influence in determining the implant of choice. It provides good-to-excellent bone healing with reduced complications and better biomechanical stability.

## Introduction

Proximal femur fractures make up 45% of all hip fractures and are the major cause of disability in the elderly [1]. The incidence of proximal femur fracture is also increasing among the young population, sustaining high energy trauma. Proximal femur fractures include the neck of the femur, inter- and subtrochanteric fractures. Both inter- and subtrochanteric fractures together are termed peritrochanteric fractures. 

Intertrochanteric fractures are classified as stable and unstable. Out of the various types, the reverse oblique fractures are a unique variety as there is an inherent tendency of medial displacement of distal fragment secondary to pull by the adductors. Hence, they are very unstable [2]. The challenging goal of any peritrochanteric fracture management is to achieve anatomic reduction with a stable fracture fixation to return the patient to his or her pre-fracture activities as soon as possible.

Over the past decades, inter- and subtrochanteric fractures were predominantly treated by implants, such as dynamic hip screw, dynamic condylar screw, angular blade plates, or cephalomedullary nails; 35-40% of these fractures are unstable three and four-part configurations with a displacement of the posteromedial cortex.

The average failure rate of these unstable fractures fixed with sliding hip screws is approximately 6-32% [3-5]. Unstable trochanteric fractures, when fixed with dynamic hip screw/dynamic condylar screw plates, cannot adequately prevent secondary limb shortening after weight-bearing due to lateralization of the head-neck fragment from gliding along the screw.

The optimal management of subtrochanteric fractures with extension into the proximal femur remains controversial. Adolescents with open physes, severely bowed or deformed femurs, and short and narrow femoral canals cannot accommodate intramedullary nails. Hence newer methods in the treatment of these fractures are critical. Many internal fixation devices have been used in the treatment of unstable proximal femur fractures because of the high incidence of complications reported after surgical treatment with each implant. Lack of satisfactory implant in the surgical treatment of these fractures has led to a series of evolution in search of a perfect implant.

The locking compression plate was introduced in the 21st century as an implant that allows angular stable plating to treat complex comminuted intertrochanteric, subtrochanteric, and osteoporotic fractures [6].

The present study was undertaken to assess the functional outcome of complex proximal femur fractures managed by the proximal femoral locking compression plate (PF-LCP).

## Materials and methods

A retrospective analysis of 30 patients with unstable peritrochanteric fractures treated with PF-LCP between 2015 and 2019 in a first-level trauma center was conducted. Inclusion criteria were the presence of an unstable peritrochanteric fracture (reverse oblique intertrochanteric/subtrochanteric fracture, lateral wall disruption), posteromedial comminution, which makes the fracture configuration unstable [7], patients aged over 18 years, with no other ipsilateral limb fracture and with a standard pre-ambulatory status. Patients with pathological fractures, polytrauma, open fractures, and pediatric cohort were excluded from the study.

Patient data were acquired from an electronic patient charting system. Fractures were classified per Arbeitsgemeinschaft fur Osteosynthesefragen (AO) 31A: 3 - proximal femur; 1A - trochanteric region; 31A1 - simple pertrochanteric; 31A2 - multifragmentary pertrochanteric lateral wall incompetent (<20.5mm); 31A3 - intertrochanteric (reverse obliquity).

In our study, 31A2 and 31A3 fractures were included. Fracture healing was defined, and postoperative status and complications were evaluated. Any discrepancies were resolved through a consensus discussion between the authors. Primary endpoints were defined as a radiological union by assessing the neck-shaft angle, postoperative complications, and functional outcomes were assessed by modified Harris Hip Score at six weeks, six months, and 12 months. The sample size was calculated to be 30. The formula for sample size calculation is as follows:

N = Z^2^ x p(1-p) \ M^2^

where N is the required sample size, Z is the confidence level at 90% (standard value of 1.645); p is the proportion of good functional outcomes in unstable proximal femur fractures (80%), and M is the margin of error at 12% (standard value of 0.12). Therefore, N = (1.645)^2^ x 0.80(1-0.80) \ (0.12)^2^**,** and thus N=30.

Data were entered in Microsoft Excel, and data analysis was done using SPSS version 19.0 (IBM Inc., Armonk, USA). Chi-square tests were done to assess the categorical variables like functional and radiological outcomes.

After the fracture was fixed with a proximal femur locking compression plate (PF-LCP), the neck-shaft angle was measured with an instantaneous postoperative radiograph. Functional outcomes were measured using Harris Hip Score. Ethics approval was obtained from the Research Ethics Board of Sundaram Medical Foundation (Dr. Rengarajan Memorial Hospital).

Bith 4.5 mm proximal femur locking plates and contralateral side distal femur locking plates were used for surgical fixation. These plates are side specific and anatomically contoured to the lateral aspect of the proximal femur. There are six distinct points of fixation within the proximal femur for stability and intraoperative versatility. These plates have a unique bullet tip, which assists with minimally invasive insertion and minimizes the prominence. Each screw hole accepts a 4.5 mm cortical and locking option, 5.7 mm cannulated locking, 6.5 mm cancellous, and also 6.5 mm cannulated locking screws accordingly. Utilizing both locking and non-locking screws, the system allows for the creation of a fixed-angle construct capable of resisting angular collapse and rotational displacement. The minimally invasive procedure is facilitated by a radiolucent targeting system designed to cut back the potential for soft tissue damage or disruption of blood supply and straightforward introduction of instruments and implants.

Surgical technique

**Figure 1:**
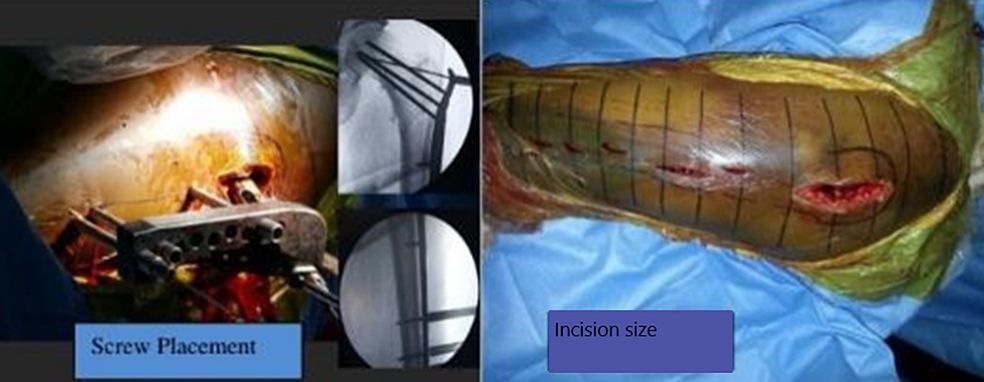
Showing jig attachment to the plate with drill bits in corresponding screw holes and required incision for the procedure

Patients were positioned supine on the fracture table. All patients received a single shot of intravenous cefazolin 2 g 30 min before the operation. A fluoroscopic image intensifier was used to guide intraoperative reduction and internal fixation. A lateral minimally invasive (Figure 1) or a traditional lateral approach was used when necessary. The fractures were reduced by proximal fixation of the angular stable screws within the head-neck fragment and so using the plate as a reduction tool to align the fractured shaft fragments. If necessary, the femoral head and neck were exposed through an anterolateral approach. It's recommended that screw insertion begins with the Alpha hole before proceeding further. Once head screws were fixed, the proper placement of the plate and screw length were ensured under fluoroscopy (Figure 1).

**Figure 2:**
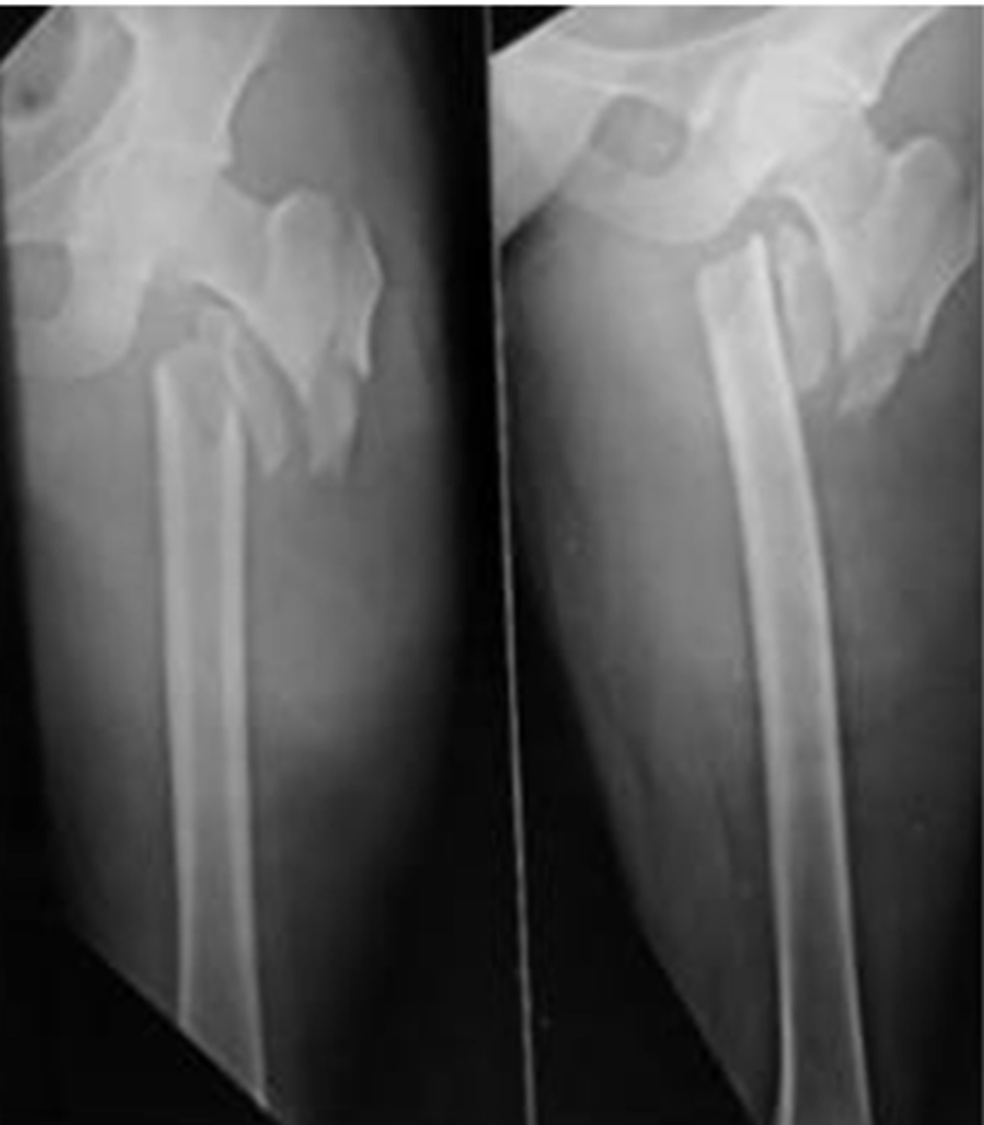
Showing unstable intertrochanteric fracture (extracapsular)

**Figure 3:**
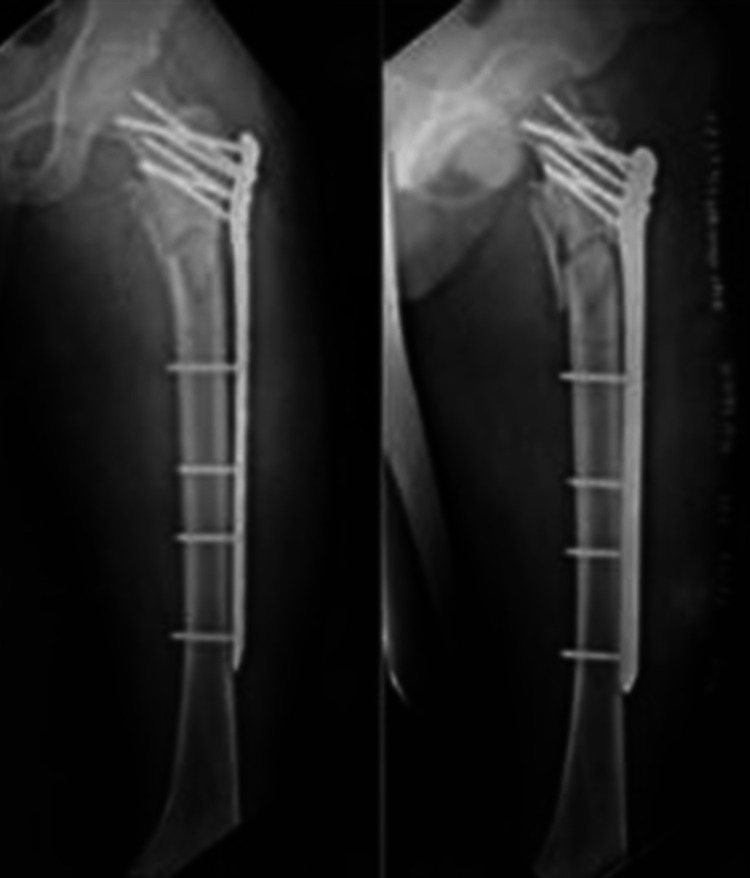
Immediate postoperative antero-posterior and lateral radiographs Fracture fixation was performed with a proximal femur locking compression plate (PF-LCP).

**Figure 4:**
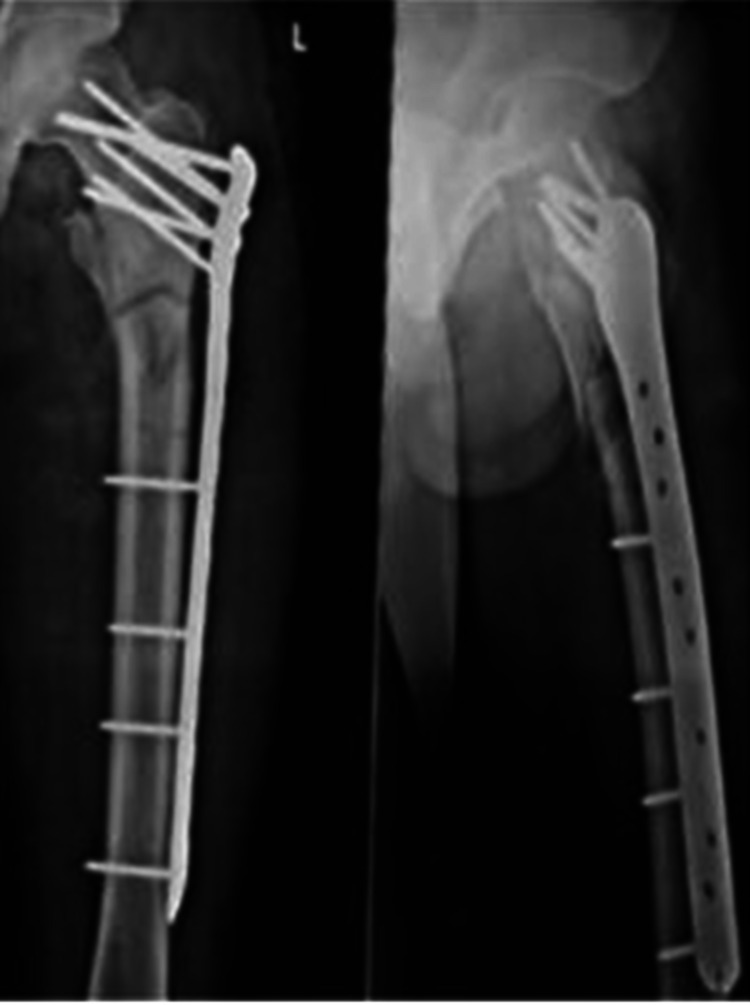
Antero-posterior and lateral radiographs at six weeks of follow-up

**Figure 5:**
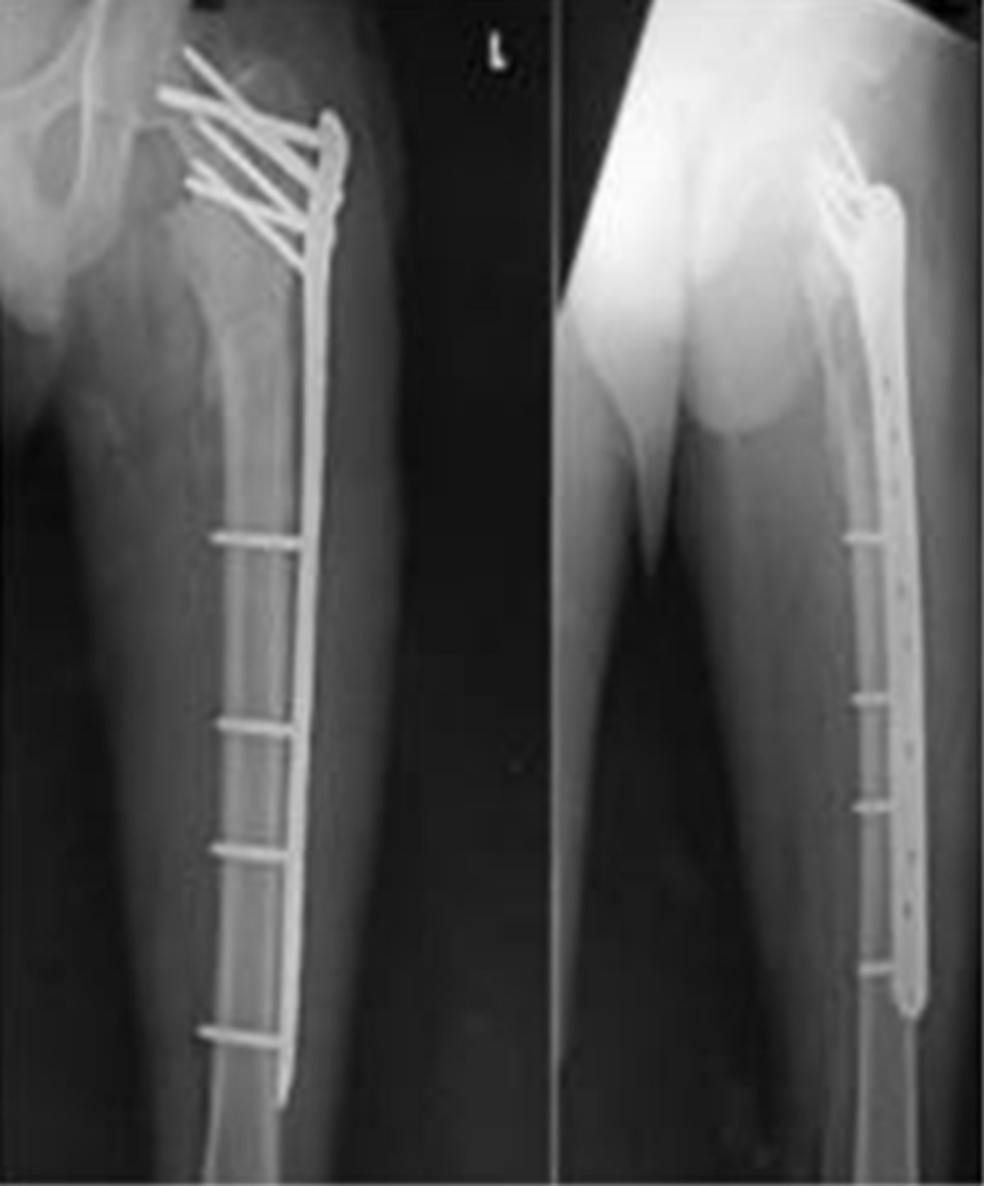
Antero-posterior and lateral radiographs at three months of follow-up

**Figure 6:**
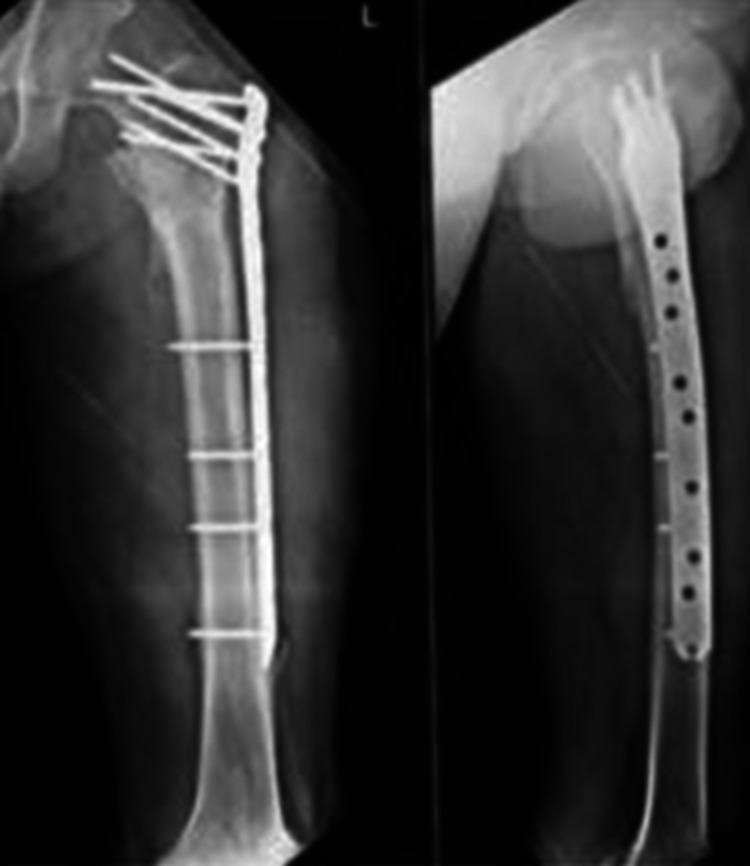
Antero-posterior and lateral radiographs showing union at the fracture site at six months of follow up

**Figure 7:**
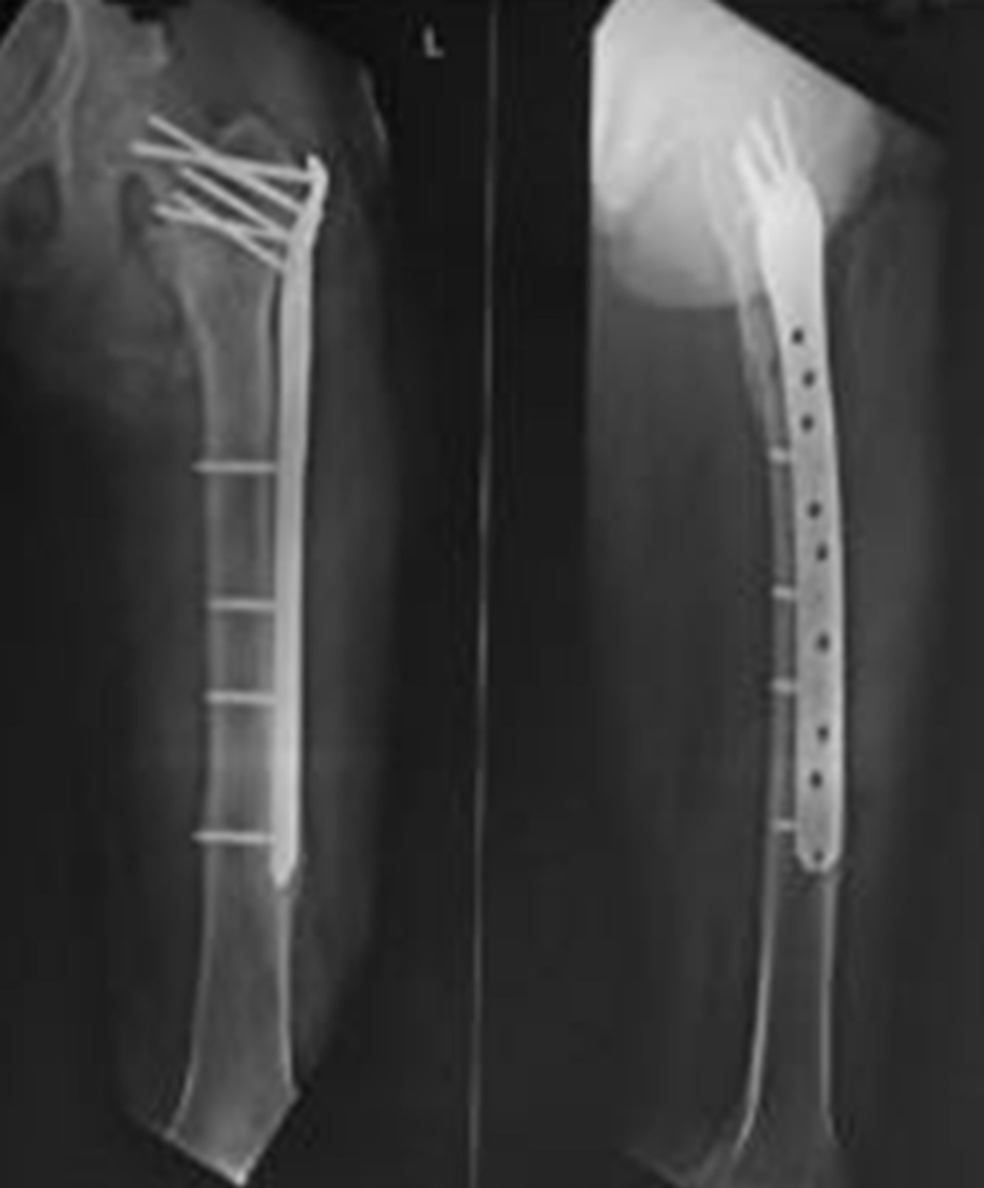
Antero-posterior and lateral radiographs at 12 months of follow up

Postoperatively, patients were allowed for partial weight-bearing (15-20 kg) from the second postoperative day. If patients weren't able to go with partial weight-bearing, wheelchair transfer was done. Further weight bearing followed in line with the clinical and radiological assessment of fracture healing. Low molecular weight heparin was administered postoperatively in prophylactic dosage until full weight-bearing was achieved. Clinical and radiological follow-ups were routinely performed at six weeks, three months, and six months. Radiological outcomes like union and the neck-shaft angles were assessed based on standard anteroposterior and lateral X-rays of the operated hip (Figures 2-7). Clinical and functional outcomes were assessed using a modified Harris Hip Score.

## Results

Thirty patients with unstable peritrochanteric fractures operated between 2015 and 2019, complying with our inclusion criteria were analyzed. All patients were operated by the same surgeon and were available for a midterm follow-up (12 months) of the functional and radiological outcomes. The mean age of the study population was 65+/-20 years, with 13 patients aged more than 70 years and three patients less than 30 years (Table 1). The most common mode of injury was trivial fall (23 patients), and a minority of the study group sustained these fractures following road traffic accident (RTA;** **n=7) as seen in Table 2. There were no perioperative complications reported in the study group. The average duration of hospital stay was 7+/-2 days. No mortality was evidenced during the study period.

**Table 1:** Age distribution

Age	Number of cases
<30yrs	3
30-40 years	1
40-50 years	1
50-60 years	4
60-70 years	8
>70 years	13
Mean age: 65+/-20 years	

**Table 2:** Mode of injury RTA - road traffic accident

Mode	Number of cases
Trivial fall	23
RTA	7

The radiological union was assessed based on the obliteration of the fracture line and the trabecular continuity of at least three out of four cortices between the two fragments in antero-posterior (AP) and lateral X-ray views. Mean radiological union time was 12.5+/-2 weeks, with 24 patients achieving union between 10-15 weeks, and three patients had a union in little more than 15 weeks. Two patients had non-union and required re-surgery, and showed signs of the union in further follow-ups (Table 3). The neck-shaft angle for 93% of patients was between 120˚-135˚ (Table 4). There were no wound-related complications. Out of the 30 patients, two patients had varus collapse with screw pullout and failed to unite, and one patient had screw pullout alone (Table 5).

**Table 3:** Union time

Union time	Number of cases
<10 weeks	1
10-15 weeks	24
>15 weeks	3
Failure of union	2
Mean union time: 12.5+/-2 weeks	

**Table 4:** Neck-shaft angle

Neck-shaft angle	Number of cases	Percentage
<120˚	2	7
120˚-135˚	28	93
>135˚	0	0

**Table 5:** Complications

Complication	Number of cases
Varus collapse with screw pullout	2
Screw pullout	1

Functional results were assessed in the 30 cases available for follow-up using Harris Hip Score. Excellent results were seen in 17 cases, good in seven cases, fair in three cases, and poor in three cases (Table 6).

**Table 6:** Functional results

Results	Number of cases
Excellent	17
Good	7
Fair	3
Poor	3

## Discussion

According to literature, 35-40% of the intertrochanteric fractures are unstable three or four-part fractures with posteromedial comminution [8]. Unstable peritrochanteric fractures remain a challenge, with no single implant receiving universal approval [9]. A major difficulty in the reduction and fixation of these fractures arises due to multiple factors, including strong deforming forces, complex fracture patterns, comminution, and very poor bone quality. Unstable fractures fixed with sliding hip screws have an approximately average 6-32% failure rate [8]. Intramedullary devices like gamma nails and proximal femoral nailing (PFN)** **have better outcomes in fixations of unstable fractures than sliding hip screws. The gamma nails had a failure rate ranging from 12.7 to 15%, based on various studies. For proximal femoral nailing, Fogagnolo et al. found that the intraoperative technical or mechanical complication rate is as high as 23.4% [10].

Stable fixation is a primary goal of treatment, thereby allowing early mobilization and restoration of limb function. It is important to improve functional outcomes and avoid complications that arise from prolonged immobilization. Internal fixation with plating offers numerous advantages over intramedullary implants, like anatomical reduction and avoiding iatrogenic compromise of abductor mechanism. The PF-LCP was developed with improvements over previous plate designs, including anatomical pre contouring to fit the proximal femur. Unlike conventional compression plate, the screw head ‘locks’ into the PF-LCP, thereby creating an angular, stable construct [6]. The PF-LCP does not fail at the screw bone interface and provides a strong anchor in osteoporotic bones [8]. The multiple locking screw holes of the PF-LCP provide multiple options to reduce and fix complex fracture patterns. The PF-LCP also functions as an internalized external fixator, and close plate-to-bone contact is not required. This minimizes damage to the periosteum and its blood supply, enabling more biological healing.

In our study, we report the radiological and functional outcomes of unstable peritrochanteric fractures fixed with the use of PF-LCP. The mean age group of our study population was 65 years, in contrast to the higher age group reported in the literature [8,11]. There was a slight female predominance (1:1.7) in contrast to the recent Indian literature [8]. Seventy-seven percent of the study population sustained the fracture by mechanical fall, similar to the statistics reported in the literature [8,12].

The minimally invasive plate osteosynthesis (MIPO) technique is used for fracture fixation (Figure 1). There were no perioperative complications reported. The majority of the patients (67%; n=20) had an acceptable reduction; a good reduction was followed in 27% (n=8) of patients and a poor reduction in 6% (n=2) of patients. On the contrary, Kovalak et al. [11] reported an 80% good reduction in their case series. The mean neck-shaft angle post radiological union in our case series was 126.1 degrees, whereas Shah et al. [8] and Kovalak et al. [11] reported post-reduction a neck-shaft angle of 125.9˚ and 129.5˚, respectively. Two of our patients had coxa vara. In our study, the mean fracture union time was 12.5 +/-2 weeks, comparatively lesser than the union time reported in the literature.

Out of 30 patients, 90% (n=27) of patients had no complications, and 6.66% (n=2) patients had varus collapse with screw pullout and progressed to non-union. Screw pullout alone was evidenced in 3.33% (n=1) of patients with minimal symptoms. Shah et al. [8], with a slightly bigger study population, reported a non-union rate of 15%, an infection rate of 30%, and other complications like coxa vara and coxa valga of 10%. Lenich et al. [ 12] in their study showed reduced varus collapses and hardware failures with the use of proximal femoral locking plate in comparison with cephalomedullary nails. Kovalak et al. [11], in their study, reported a 6.45% (n=2) infection rate, screw pullout in two patients in their study population. Lee et al. [13], in their study, had loosening of locking screws in 15.3% (n=4), delayed union in 7.6% (n=2), and one patient sustained deep infection.

In our study, 6.66% (n=2) patients underwent re-operation due to impending non-union. On the contrary, Kumar et al. [14] reported one patient having re-operation, and Halder et al. [15] reported 25% (n=4) patients had re-operation. A modified Harris Hip Score was used to assess the functional outcome and the mean score was 84.26. Overall, good to excellent results were seen in 80% of cases, excellent in 57% (n=17) patients, good in 23% (n=7) patients, fair in 10% (n=3) patients and poor in 10% (n=3) patients. The results obtained were slightly lower in comparison to Hu et al. [16] series, which reported a mean Harris Hip Score of 86.5. The functional outcomes of our study population were comparatively better than those reported by Shah et al. [8] (80.2) and Kovalak et al. [11] (78.24).

Limitations of this study are mainly small sample size, short term follow-up period, no alternative treatment/control group included in this study to compare these results; thus, we had to compare our results with available literature.

## Conclusions

The application of the PF-LCP is also justified for its superior abilities to revive and maintain anatomy and biomechanical stability. This especially holds true for a younger subgroup of patients. However, if an anatomical reduction is not achieved and patient compliance is low, the usage of a PF-LCP should be carefully weighed against other implants, especially in unstable intertrochanteric/subtrochanteric fractures. Hence PF-LCP is a feasible alternative to treat peritrochanteric fractures. The PF-LCP is suitable for complex and unstable proximal femoral fractures with poor bone quality, lateral wall comminuted trochanteric fractures, and multifragmentary subtrochanteric fractures.
